# Investigating the experience of transgender inmates: a systematic review of qualitative studies

**DOI:** 10.1192/j.eurpsy.2025.2419

**Published:** 2025-08-26

**Authors:** M. Marchi, E. Vaccari, I. Corbellini, S. Ferrari, L. Ghirotto, G. M. Galeazzi

**Affiliations:** 1 Department of Biomedical, Metabolic and Neural Sciences, University of Modena and Reggio Emilia, Modena; 2 Qualitative Research Unit, AUSL-IRCCS Reggio Emilia, Reggio Emilia, Italy

## Abstract

**Introduction:**

Research on transgender issues has significantly advanced in recent years, yet transgender individuals in correctional facilities (CF) remain an overlooked and vulnerable population. Transgender inmates (TGI) within CF face intersectional and systemic challenges, particularly concerning housing placement, access to gender-affirming treatments, and mental health support.

**Objectives:**

This systematic review of qualitative studies aims to explore the experience of TGI in CF.

**Methods:**

Medline, Embase, Scopus, PsychInfo, and CINAHL were searched for studies on TGI in CF published until 31 December 2023. Qualitative studies capturing the voices of TGI detained in CF at the time of data collection were eligible for inclusion. Two independent reviewers extracted key constructs (researcher interpretations) and narratives (participant voices), categorizing them into labels and analytical themes. Intensity (I) and frequency (F) were calculated for each theme as the proportion of labels and studies associated with that theme, respectively. Following a socio-constructivist approach, a meta-synthesis was performed to create a new conceptual framework capturing the subjective experiences of TGI in CF. The study was registered with PROSPERO, CRD42023456340.

**Results:**

From 641 studies initially identified, 33 full texts were analyzed, leading to a selection of 13 qualitative studies of TGI in CF. Ten main themes emerged, based on 537 coded labels: (1) feelings and identity (F:92.3%, I:29.1%); (2) mental and physical violence (F:84.6%, I:12.5%); (3) social issues and connections (F:76.9%, I:11.0%); (4) housing (F:76.9%, I:10.2%); (5) mental and physical health (F:61.5%, I:8.8%); (6) discrimination (F:53.8%; I:6.9%); (7) therapy (F:46.1%, I:6.2%); (8) definition and language (F:38.4%, I:11.6%); (9) coming out (F:38.4%, I:2.6%); (10) expectations (F:15.3%, I:1.3%). These findings inform the proposed Safety Model, a new framework for understanding TGI experiences in CF.

**Conclusions:**

CF, as a total institution, with binary and heteronormative characteristics, often fails to ensure the safety of TGI. This review underscores incarceration as a significant social determinant of health for TGI. A comprehensive policy reform and targeted staff training should be promoted to foster inclusive and supportive correctional environments and improve the safety and health of TGI.

**Disclosure of Interest:**

None Declared

